# Network pharmacology analysis reveals potential targets and mechanisms of proton pump inhibitors in breast cancer with diabetes

**DOI:** 10.1038/s41598-023-34524-x

**Published:** 2023-05-10

**Authors:** Haihong Hu, Hanbin Wang, Xiaoyan Yang, Zhicheng Li, Wendi Zhan, HongXia Zhu, Taolan Zhang

**Affiliations:** 1 Department of Pharmacy, The First Affiliated Hospital, Hengyang Medical School, University of South China, Hengyang, 421000 Hunan China; 2 School of Pharmacy, Hengyang Medical College, University of South China, 28 Western Changsheng Road, Hengyang, 421001 Hunan China; 3 Chinese Traditional Medicine (CTM) Research Platform of Major Epidemic Treatment Base, The First Affiliated Hospital, Hengyang Medical School, University of South China, Hengyang, 421000 Hunan China

**Keywords:** Cancer, Cell biology, Computational biology and bioinformatics, Drug discovery, Molecular biology, Oncology, Pathogenesis

## Abstract

Breast cancer and diabetes are significant health challenges, and effective treatments for both diseases are lacking. Proton pump inhibitors (PPIs) have demonstrated anticancer and hypoglycemic effects, but their mechanisms of action are not yet fully understood. We used the GeneCards and PharmMapper databases to identify therapeutic targets for diabetes,  breast cancer and PPIs. We identified common targets and constructed a regulatory network of diseases and drugs using the STRING database and Cytoscape software. We also explored the binding between small molecule ligands and protein receptors using Discovery Studio software. We identified 33 shared targets for breast cancer, diabetes, and PPIs including lansoprazole, omeprazole, and pantoprazole, which play a critical role in fatty acid transport, insulin resistance, apoptosis, and cancer-related signaling pathways. Our findings demonstrated that PPIs had a strong affinity for *AKT1* and *MMP9*. This study provides insights into the mechanisms of action of PPIs in breast cancer and diabetes and identifies *AKT1* and *MMP9* as critical targets for future drug development. Our findings highlight the potential of PPIs as a novel therapeutic approach for these challenging diseases.

## Introduction

Breast cancer, a malignant tumor that occurs in breast tissue, has become a disease that seriously affects women’s health. According to the data released by the International Agency for Research on Cancer (IARC) of the World Health Organization (WHO) in 2018, the incidence of breast cancer among women is 24.2%, and the mortality rate has reached 15%, which has become the first death-associated cancer in women^1^. Although clinical treatment for breast cancer has been effective, the recurrence of breast cancer after surgery and the emergence of drug resistance during treatment still pose a great challenge to breast cancer treatment^2,3^. Although the specific pathogenesis of breast cancer has not been fully elucidated, with the deepening of breast cancer research^4^, some risk factors closely related to the occurrence and development of breast cancer have been found^5^. Among them, diabetes mellitus has been widely noticed due to its potential association with the development of some tumors^6^. Recent epidemiological studies have shown a positive association between diabetes and the risk of breast cancer development and death^7,8^. Glucose in diabetes has been thought to be a risk factor for breast cancer in previous studies, but current evidence suggests that hyperglycemia promotes tumor cell growth only in the presence of insulin^9,10^. However, because the complex pathogenesis between the two diseases has not been elucidated, there is no effective drug available for the treatment of breast cancer patients with diabetes. Together with the yearly increase in the incidence of breast cancer and diabetes and the young age of the incidence population, which makes treatment of breast cancer and diabetes become a great challenge at present.

Proton pump inhibitors (PPIs) are a class of drugs primarily used to treat gastric acid-related gastrointestinal conditions, such as peptic ulcers, Helicobacter pylori infections, and gastroesophageal reflux disease^11^. However, such a classic old drug has attracted extensive attention again due to its special efficacy in other diseases^12,13^. Multiple studies have shown that PPIs are associated with reduced cancer risk and may play a positive role in the treatment of other malignancies, which arouses interest in the field of oncology^14,15^. PPIs can increase the sensitivity of breast cancer cells to chemotherapy and radiotherapy drugs, which may be closely related to their acidic tumor microenvironment^16^. Acidification of the tumor microenvironment is a hallmark of malignancy and plays a very important role in tumor cell appreciation, invasion, metastasis, and drug resistance^17,18^. The survival of tumor cells in this acidic environment depends on a number of proton exchangers, including vesicular ATPase (V-ATPase), which maintains the normal physiological activities of tumor cells by promoting extracellular acidification by removing intracellular H^+^ from the cell^19,20^. In addition, recent studies have revealed the mechanism of the hypoglycemic effect of PPIs, such as improving insulin resistance and increasing insulin secretion^21^. For instance, PPIs have been found to improve insulin resistance and increase insulin secretion, leading to lower blood sugar levels and increased insulin utilization^21,22^. However, the exact mechanism of PPIs in breast cancer and diabetes remains unclear and requires further study.

**Figure 1:**
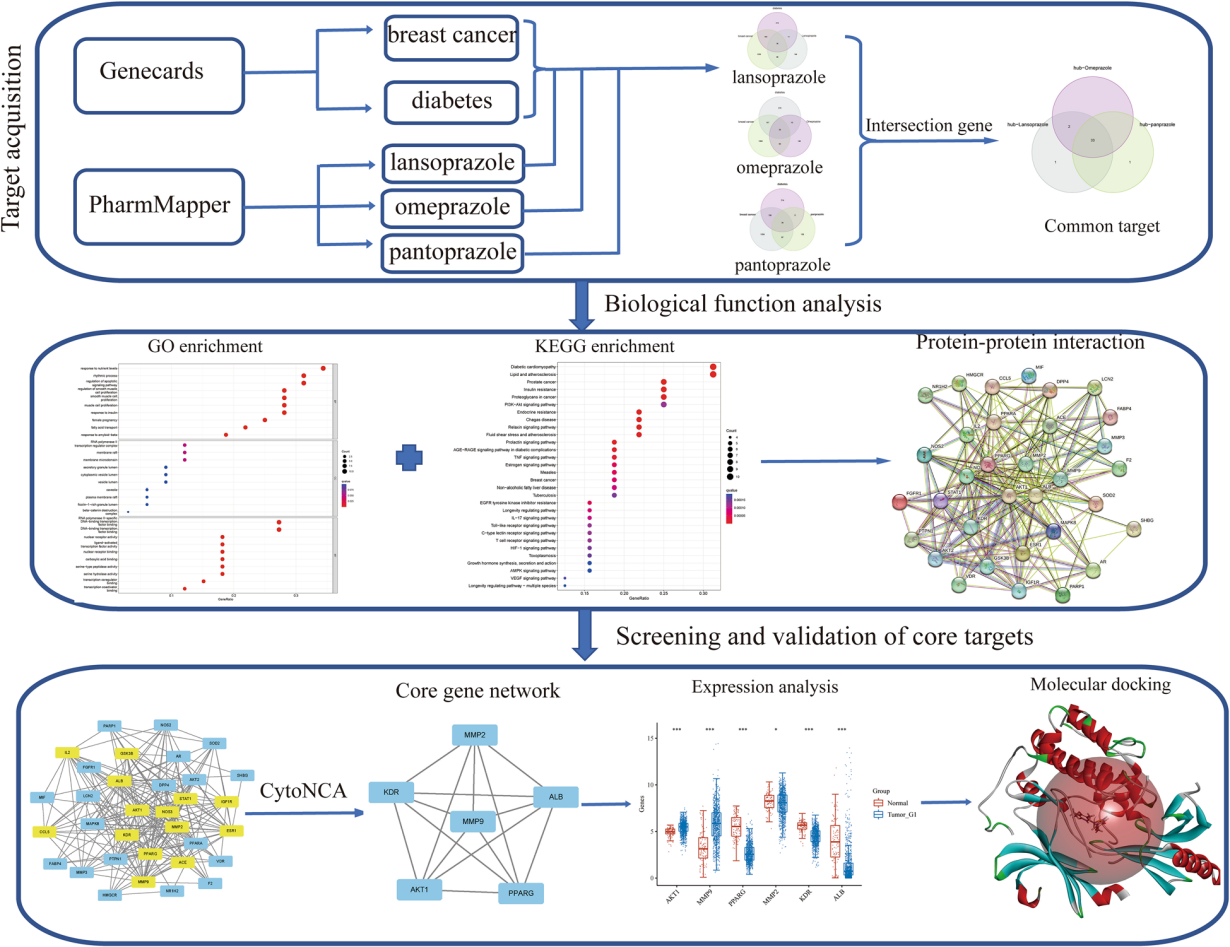
Flow chart of network pharmacology to investigate the potential molecular mechanisms of proton pump inhibitors in the treatment of breast cancer and diabetes.

The potential use of PPIs in the treatment of breast cancer and diabetes has been a subject of interest. To better understand their mechanism of action and potential therapeutic targets, this study utilized network pharmacology and molecular docking methods with dual diseases as a starting point (refer to Fig. 1). The study identified 33 common targets of PPIs at the intersection of breast cancer and diabetes, where *AKT1* and *MMP9* were found to be the core targets. Moreover, the study also highlighted the significant role of fatty acids in breast cancer and diabetes, which could provide a theoretical foundation for the development of new drugs and the investigation of the underlying mechanisms of these diseases.

## Materials and methods

### Collection of the intersection genes of breast cancer, diabetes, and proton pump inhibitors

Therapeutic targets that were filtered for a correlation score greater than 10 for breast cancer and diabetes were obtained directly from the GeneCards database (https://www.genecards.org/)^23^. To identify potential targets and molecular mechanisms, we used pharmacophores from small molecule results to predict the target sites. We used PharmMapper (http://lilab-ecust.cn/pharmmapper/index.html) to obtain the predicted targets of representative drugs of proton pump inhibitors including lansoprazole, omeprazole, and pantoprazole^24,25^. Then the target was annotated by Perl software (https://www.perl.org/, version 5.34.1) to convert the serial number into a specific gene symbol. This approach allowed us to identify potential therapeutic targets for breast cancer and diabetes and gain insights into the molecular mechanisms underlying these diseases.

### Common target of proton pump inhibitors in the treatment of dual diseases

Subsequently, we looked for the predicted targets of lansoprazole, omeprazole, and pantoprazole and the common targets of genes related to breast cancer and diabetes respectively, and then took the common target genes of three kinds of drugs as our research objects. We used the intersect function in R software (version 4.0.3) which was a free and open-source programming language and software environment for statistical computing and graphics to screen the common targets of breast cancer, diabetes and proton pump inhibitor representative drugs and invoked the R package Venn to visualize the results of intersection genes and output the intersection genes.

### GO and KEGG enrichment analysis

Enrichment analysis was conducted to identify significantly enriched biological processes and signaling pathways among the intersection of drug and disease targets. Firstly, the gene symbols were converted to Ensembl IDs using the org.Hs.eg.db package. Next, Gene Ontology (GO) analysis was performed using the “enrichGO” function in the R package clusterProfiler, and Kyoto Encyclopedia of Genes and Genomes (KEGG) pathway analysis was performed using the “enrichKEGG” function^26^. To filter for enriched categories or pathways, a significance threshold of *p* value < 0.05 and q-value < 0.05 was applied, and the degree of enrichment was represented using the − log10 transformed *p* value. Categories with a *p* value and q-value less than 0.05 were considered significant. The top 10 enriched results were visualized for both KEGG analysis and the three modules of GO enrichment analysis, which included biological processes (BP), cellular components (CC), and molecular functions (MF). The enriched categories and pathways identified in this study provide insights into the biological mechanisms underlying the drug and disease targets and may help in the identification of potential therapeutic targets.

### Construction of a regulatory network and screening of core hub genes

To construct a comprehensive regulatory network for crossover genes, we utilized the STRING online database with a medium confidence threshold set at > 0.4 to obtain protein–protein interaction information^27^. Subsequently, we imported the interaction data into Cytoscape software version 3.9.1 to visualize the regulatory network of diseases, drugs, and signaling pathways^28^. To identify key components in the network, we used the CytoNCA tool in Cytoscape, which analyzed the network data based on various criteria such as Betweenness, Closeness, Degree, Eigenvector, LAC, and Network^29^. Subsequently, transcriptome information of 459 normal samples from GTEX and 113 normal samples from TCGA and 1101 patients with breast cancer was collected to study the differential expression of the above six core genes. *AKT1* and *MMP9* were finally selected as potential therapeutic targets. This filtering approach allowed us to identify the most important and influential nodes in the network, which could play a critical role in the pathogenesis of the studied diseases or serve as potential therapeutic targets.

### Molecular docking for ligand–protein interaction prediction

The 3D structure of the small molecule ligand was obtained from PubChem (https://pubchem.ncbi.nlm.nih.gov/), and the protein structure was downloaded from the PDB (https://www.rcsb.org/) database (*AKT1*: 4ejn; *MMP9*: 6esm). The ligand was optimized using the Prepare Ligand module in Discovery Studio (v19.1.0) with default parameters. The Gasteiger–Marsili method was used to calculate the partial charges. The force field used for the optimization was CHARMm. Molecular docking simulation was performed using the CDOCKER algorithm in Discovery Studio. To ensure the accuracy of the selected docking method, the root mean square deviation (RMSD) was calculated between the co-crystal ligand and the prepared ligand structure using the Calculate RMSD function in Discovery Studio. The calculated RMSD value was 0.5274 Å (*AKT1*) and 0.9494 Å (*MMP9*), indicating that the docking method was suitable for predicting the binding modes of the ligands. The grid box was defined to enclose the active site of the protein with the XYZ coordinates of *AKT1* (35.389442, 43.721140, and 18.443372) and *MMP9* (1.781333, 50.977267 and 19.670200), and the radius was set to 15.743137 (*AKT1*) and 11.258874 (*MMP9*). The ligands were docked into the active site of the protein using the CDOCKER algorithm with default parameters. The number of runs was set to 10, and the best pose of each ligand was selected based on the CDOCKER interaction energy. The binding free energy was calculated after docking using the Calculate Binding Free Energy module in Discovery Studio.

## Results

### Acquisition of co-targets for proton pump inhibitors and diseases

**Figure 2:**
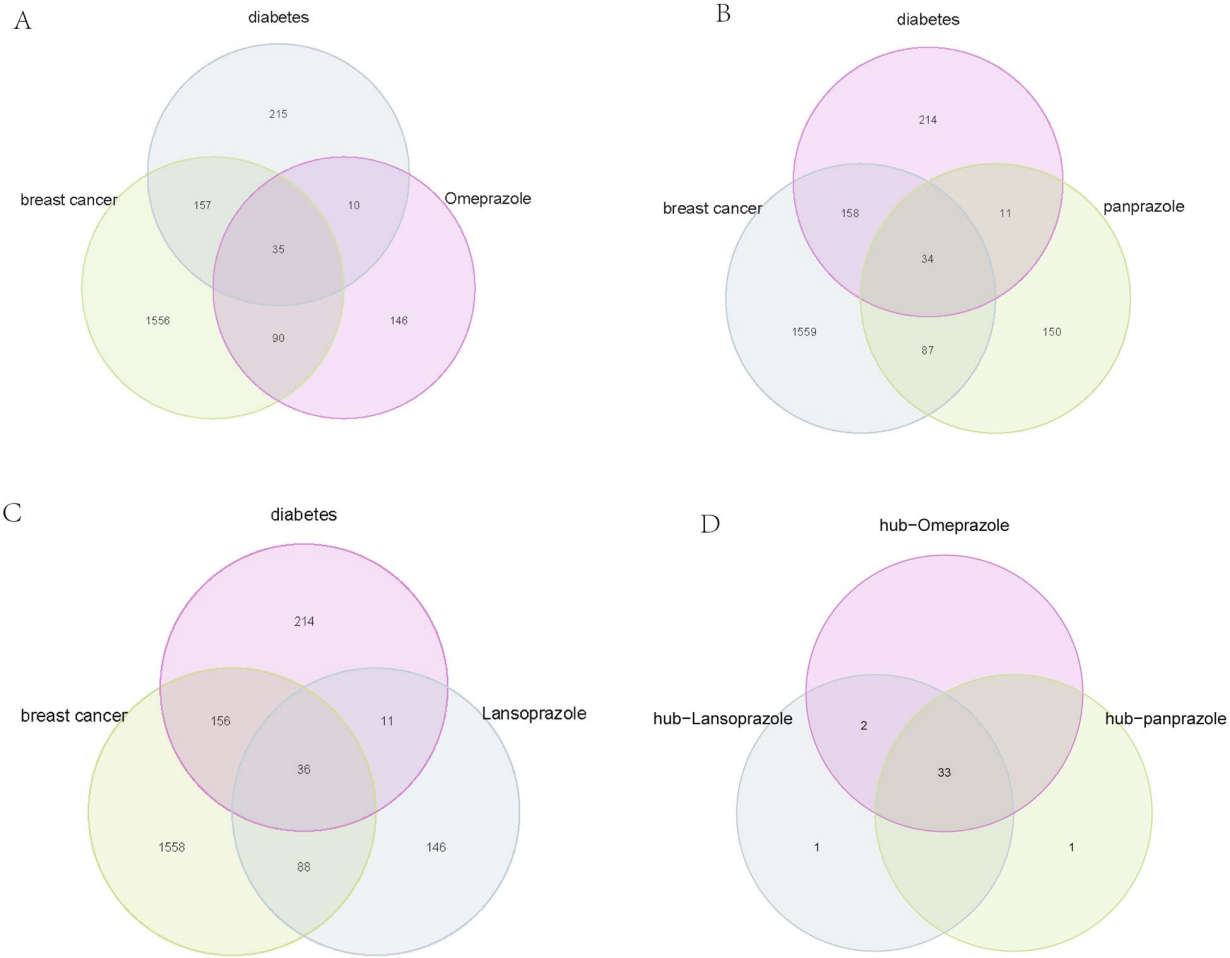
Identification of Common Targets of Proton Pump Inhibitors and Breast Cancer and Diabetes. (**A**) Venn diagram showing the 35 common targets of omeprazole and breast cancer and diabetes. (**B**) Venn diagram showing the 34 common targets of pantoprazole and breast cancer and diabetes. (**C**) Venn diagram showing the 36 common targets of lansoprazole and breast cancer and diabetes. (**D**) Venn diagram showing the 33 intersection genes identified as common targets of proton pump inhibitors and the two diseases.

In this study, a total of 1838 targets for breast cancer and 417 targets for diabetes were obtained from the GeneCards database using a relevance score of ≥ 10. The 2D structures of omeprazole, lansoprazole, and pantoprazole were downloaded from PubChem and corresponding predicted targets were obtained from the PharmMapper database, which uses three major pharmacophore groups to make target predictions. The predicted targets of the three proton pump inhibitors were intersected with the disease targets, resulting in the identification of 35 common targets of omeprazole and breast cancer and diabetes (Fig. 2A), 34 common targets of pantoprazole and breast cancer and diabetes (Fig. 2B), and 36 common targets of lansoprazole and breast cancer and diabetes (Fig. 2C). Finally, 33 intersection genes were identified as common targets of proton pump inhibitors and the two diseases (Fig. 2D).

### Enrichment analysis identifies key biological processes and pathways related to proton pump inhibitor targets

**Figure 3:**
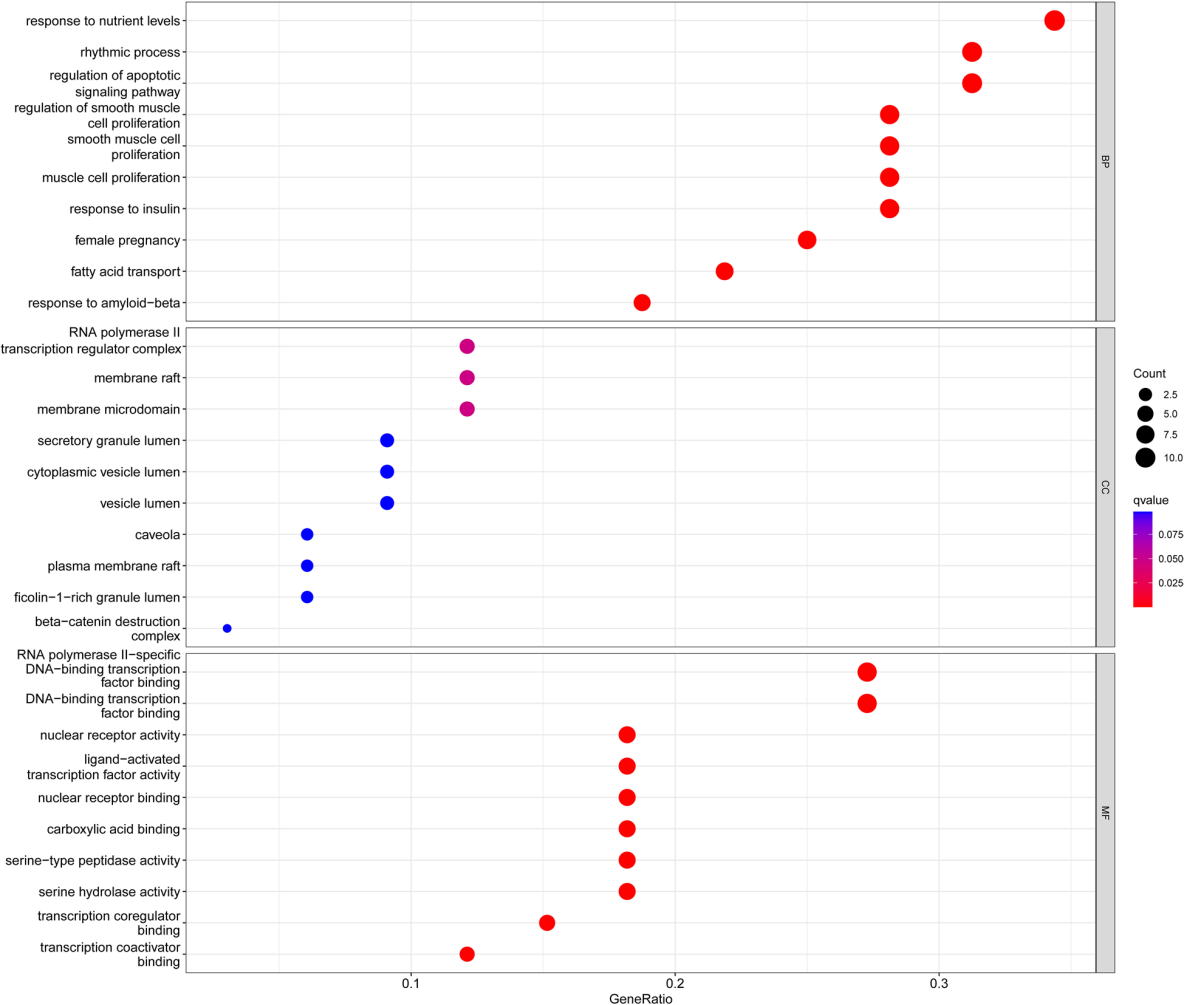
The results of GO enrichment analysis involving biological processes (BP), cellular components (CC), and molecular functions (MF).

**Figure 4:**
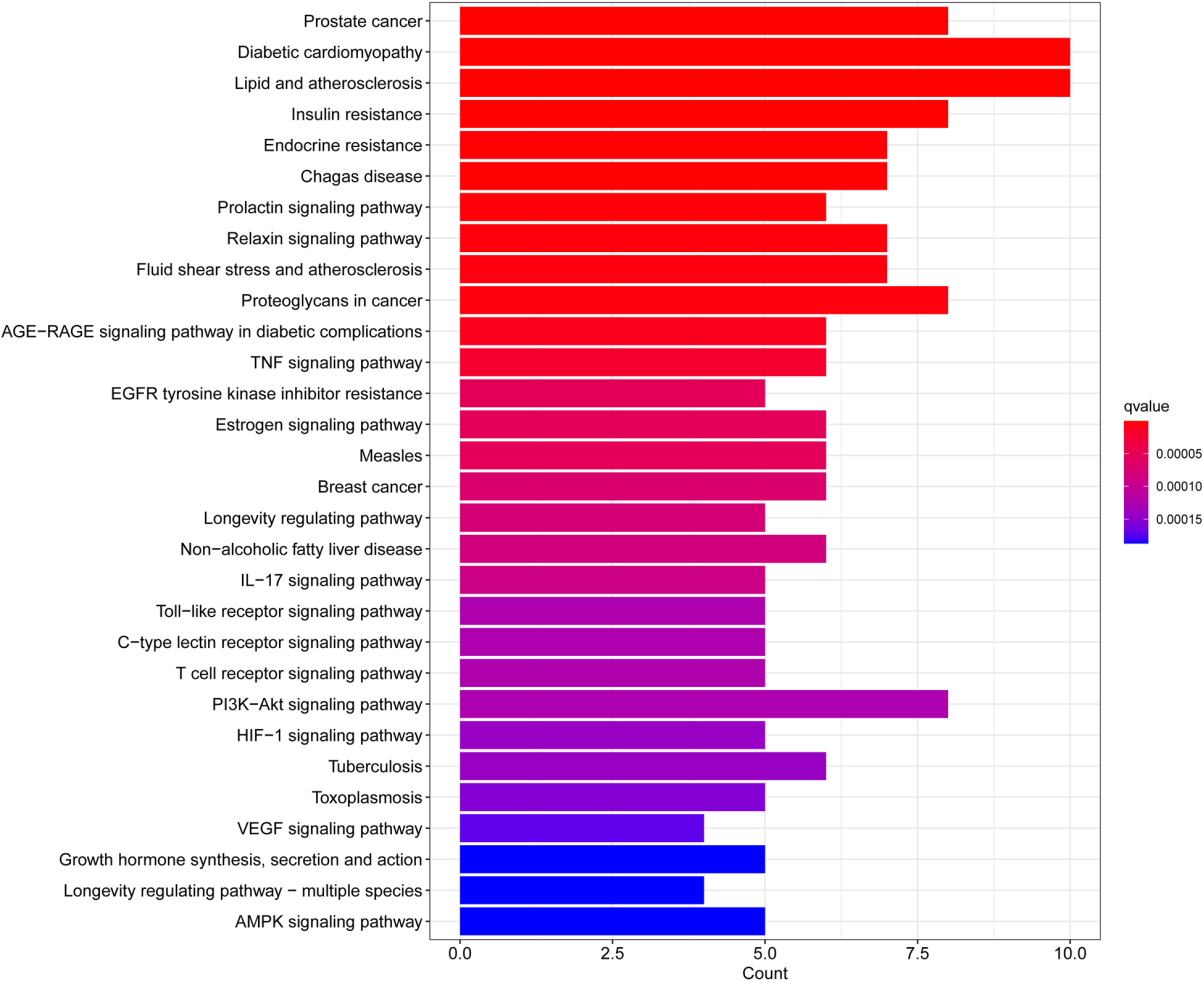
The results of Kyoto Encyclopedia of Genes and Genomes (KEGG) enrichment analyses.

After obtaining the common targets of proton pump inhibitors and diseases, we performed GO and KEGG enrichment analyses to explore the biological processes involved in the identified genes. We investigated the overrepresented biological processes and pathways using gene ontology (GO) and Kyoto Encyclopedia of Genes and Genomes (KEGG) enrichment analyses. The GO enrichment analysis revealed several significantly enriched biological processes (BP), molecular functions (MF), and cellular components (CC) among the differentially expressed genes. The top ten enriched GO terms in each category are shown in Table 1. From the perspective of biological processes, the therapeutic targets were mainly enriched in fatty acid transport, cell proliferation, and regulation of apoptotic signaling, metabolic processes of small molecules, responses to reactive oxygen species, etc. However, for cellular components, there was no significant enrichment of target genes (*p* > 0.05). For molecular functions, the targets were mostly enriched in the activity of nuclear receptors, ligand − activated transcription, nuclear hormone receptors (Fig. 3). The KEGG pathway analysis revealed several significantly enriched pathways among the differentially expressed genes, including prostate cancer, diabetic cardiomyopathy, lipid and atherosclerosis, insulin resistance. The top ten enriched pathways are shown in Table 2. These pathways are known to play important roles in Signaling pathways associated with the pathogenesis of diabetes and cancer development and progression (Fig. 4). Overall, the GO and KEGG enrichment analyses provided insights into the biological processes and pathways that are dysregulated in the studied condition and could provide potential therapeutic targets for further investigation.

**Table 1:** The top ten results of GO enrichment analysis.

	ID	Description	*p* value	*p* adjust	q value
BP	GO:0015908	Fatty acid transport	4.81E−12	5.70E−09	2.47E−09
BP	GO:0033002	Muscle cell proliferation	5.47E−12	5.70E−09	2.47E−09
BP	GO:0048660	Regulation of smooth muscle cell proliferation	8.70E−12	5.70E−09	2.47E−09
BP	GO:0048659	Smooth muscle cell proliferation	9.65E−12	5.70E−09	2.47E−09
BP	GO:2001233	Regulation of apoptotic signaling pathway	4.14E−11	1.95E−08	8.48E−09
BP	GO:0062012	Regulation of small molecule metabolic process	1.19E−10	4.57E−08	1.98E−08
BP	GO:0000302	Response to reactive oxygen species	1.35E−10	4.57E−08	1.98E−08
BP	GO:0062197	Cellular response to chemical stress	2.51E−10	7.41E−08	3.22E−08
BP	GO:0034614	Cellular response to reactive oxygen species	3.22E−10	8.46E−08	3.67E−08
BP	GO:0015718	Monocarboxylic acid transport	3.88E−10	9.16E−08	3.98E−08
MF	GO:0004879	Nuclear receptor activity	4.01E−10	4.01E−08	1.77E−08
MF	GO:0098531	Ligand-activated transcription factor activity	4.01E−10	4.01E−08	1.77E−08
MF	GO:0061629	Rna polymerase ii-specific dna-binding transcription factor binding	1.83E−08	1.19E−06	5.27E−07
MF	GO:0051427	Hormone receptor binding	2.38E−08	1.19E−06	5.27E−07
MF	GO:0140297	Dna-binding transcription factor binding	1.39E−07	5.54E−06	2.45E−06
MF	GO:0035257	Nuclear hormone receptor binding	1.96E−07	6.52E−06	2.88E−06
MF	GO:0051721	Protein phosphatase 2a binding	3.01E−07	8.59E−06	3.80E−06
MF	GO:0008236	Serine-type peptidase activity	9.10E−07	1.88E−05	8.31E−06
MF	GO:0016922	Nuclear receptor binding	9.60E−07	1.88E−05	8.31E−06
MF	GO:0017171	Serine hydrolase activity	1.03E−06	1.88E−05	8.31E−06

**Table 2:** The top ten results of KEGG enrichment analysis.

	ID	Description	*p* value	*p* adjust	q value
KEGG	hsa05215	Prostate cancer	2.22E−09	2.24E−07	8.21E−08
KEGG	hsa05415	Diabetic cardiomyopathy	2.56E−09	2.24E−07	8.21E−08
KEGG	hsa05417	Lipid and atherosclerosis	4.46E−09	2.30E−07	8.45E−08
KEGG	hsa04931	Insulin resistance	5.27E−09	2.30E−07	8.45E−08
KEGG	hsa01522	Endocrine resistance	6.96E−08	2.44E−06	8.94E−07
KEGG	hsa05142	Chagas disease	9.20E−08	2.68E−06	9.84E−07
KEGG	hsa04917	Prolactin signaling pathway	2.25E−07	5.62E−06	2.06E−06
KEGG	hsa04926	Relaxin signaling pathway	4.63E−07	1.01E−05	3.72E−06
KEGG	hsa05418	Fluid shear stress and atherosclerosis	7.70E−07	1.37E−05	5.02E−06
KEGG	hsa05205	Proteoglycans in cancer	7.82E−07	1.37E−05	5.02E−06

### Identification of specific targets of proton pump inhibitors via PPI network analysis

**Figure 5:**
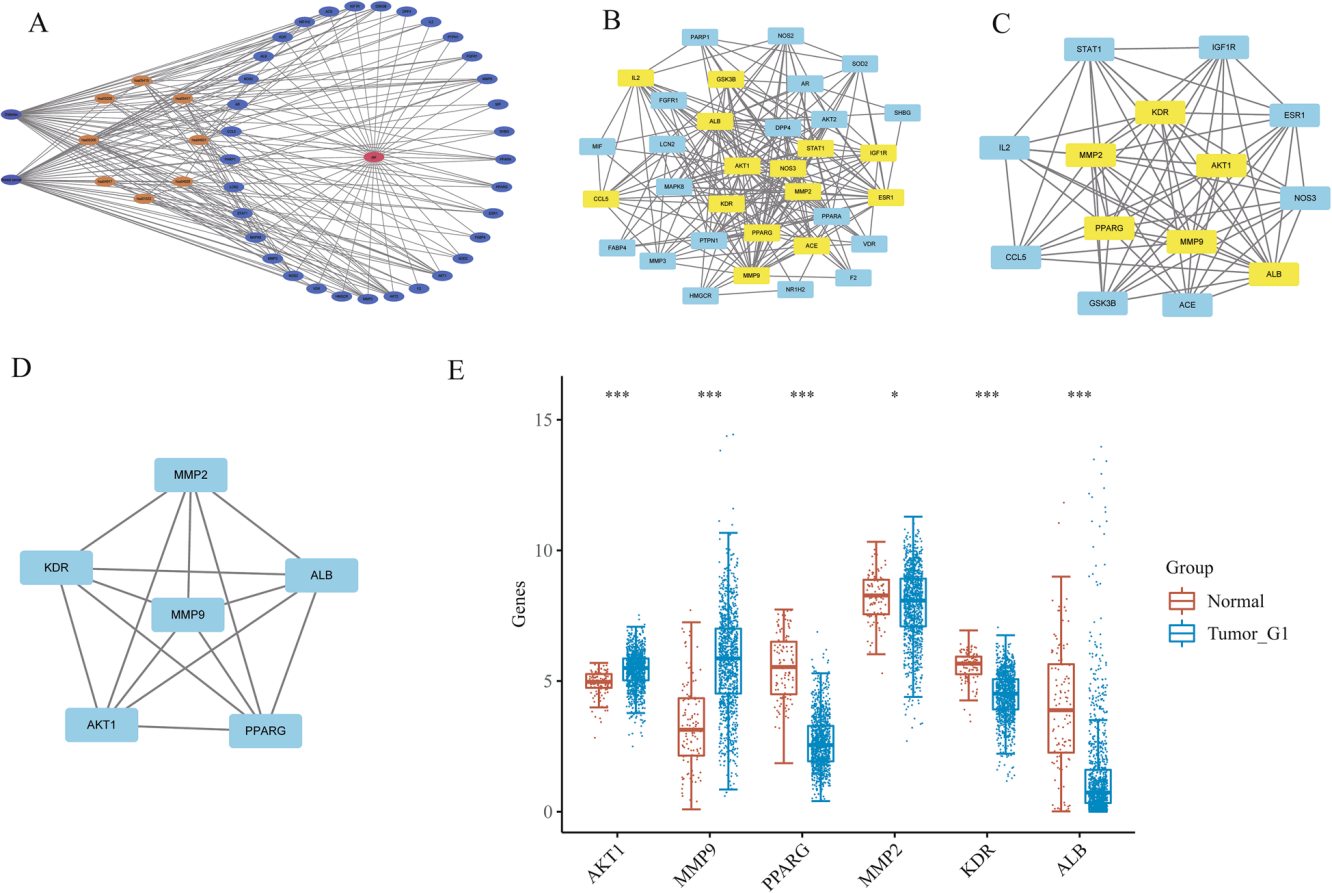
(**A**) Network of the disease-target-pathway relationship. (**B**–**D**) A core component of a regulatory network. (**E**) Differential expression of core genes in normal tissues and breast cancer.

After constructing the regulatory network using the STRING database and Cytoscape software, we obtained scores for each node using the CytoNCA tool, which calculates various network centrality measures including betweenness, closeness, degree, eigenvector, LAC, and network. The scores of the 33 targets are shown in Table 3. The degree score indicates the number of edges that connect to a node, which represents the importance of the target in the network. The betweenness score reflects the extent to which a target serves as a bridge in the network. The closeness score measures the distance between a target and all other targets in the network. The eigenvector score reflects the connectivity of a target with other important targets in the network. The LAC score measures the extent to which a target is clustered in the network. The network score reflects the overall importance of a target in the network. We used the median value of each score to filter the targets twice, resulting in six core targets: *AKT1*, *MMP9*, *PPARG*, *MMP2*, *KDR*, and *ALB* (Fig. 5A–D). To further investigate their potential as therapeutic targets, we analyzed the expression levels of these targets in breast cancer and normal tissues. Our analysis showed that only *AKT1* and *MMP9* were significantly upregulated in breast cancer tissues compared to normal tissues, suggesting that they may be the most promising targets for follow-up research (Fig. 5E).

**Table 3:** Every node in regulatory networks.

Name	Betweenness	Closeness	Degree	Eigenvector	LAC	Network
ACE	17.3877	0.653061	15	0.182951	8.266667	11.09683
MMP2	27.03777	0.711111	19	0.228283	10.31579	15.18183
KDR	10.63402	0.653061	15	0.196652	9.466667	11.37873
PPARG	80.25624	0.780488	23	0.252869	10	18.45294
HMGCR	5.546429	0.561404	7	0.094354	4.571429	6
ESR1	36.93997	0.695652	19	0.216529	9.368421	14.97323
VDR	0.944444	0.561404	7	0.102349	4.857143	5.666667
MMP3	2.221429	0.581818	10	0.137965	7.4	8.380952
PPARA	36.57475	0.666667	16	0.175828	7	10.33621
LCN2	3.036111	0.571429	8	0.103559	5.25	6.214286
AKT1	109.2935	0.914286	29	0.306216	11.72414	27.85847
F2	0	0.551724	6	0.093636	5	6
MMP9	51.77641	0.8	24	0.275692	11.66667	21.44518
DPP4	3.575469	0.603774	11	0.15578	7.454545	8.2
NOS3	19.63896	0.695652	18	0.220003	10.11111	14.28977
ALB	150.8895	0.941176	30	0.30613	11.2	28.75118
MIF	0	0.533333	5	0.078692	4	5
IL2	15.95523	0.653061	15	0.182106	8.4	11.02483
FABP4	0.883333	0.542373	5	0.073067	2.8	3.5
IGF1R	8.932684	0.64	14	0.182502	8.857143	10.39161
GSK3B	9.186003	0.64	14	0.183653	8.857143	10.45865
NOS2	4.353247	0.615385	12	0.162437	8.333333	9.333333
STAT1	10.49769	0.653061	15	0.193762	9.2	10.85723
PARP1	1.980159	0.561404	8	0.107677	5.25	6
PTPN1	8.312049	0.615385	12	0.14747	6.666667	7.737374
AR	5.702381	0.581818	10	0.129546	6.8	8.492063
AKT2	1.812771	0.592593	10	0.143848	7.4	8.222222
MAPK8	7.417677	0.64	14	0.189385	9.142857	10.41647
FGFR1	0.388889	0.542373	6	0.089241	4.333333	5.2
SOD2	4.122294	0.592593	10	0.140458	6.4	7.206349
CCL5	8.569517	0.627451	13	0.164518	8.307692	10.30418
SHBG	0	0.516129	3	0.041461	2	3
NR1H2	0.133333	0.470588	3	0.033444	1.333333	2

### Molecular docking analysis of proton pump inhibitors with *AKT1* and *MMP9*

**Figure 6:**
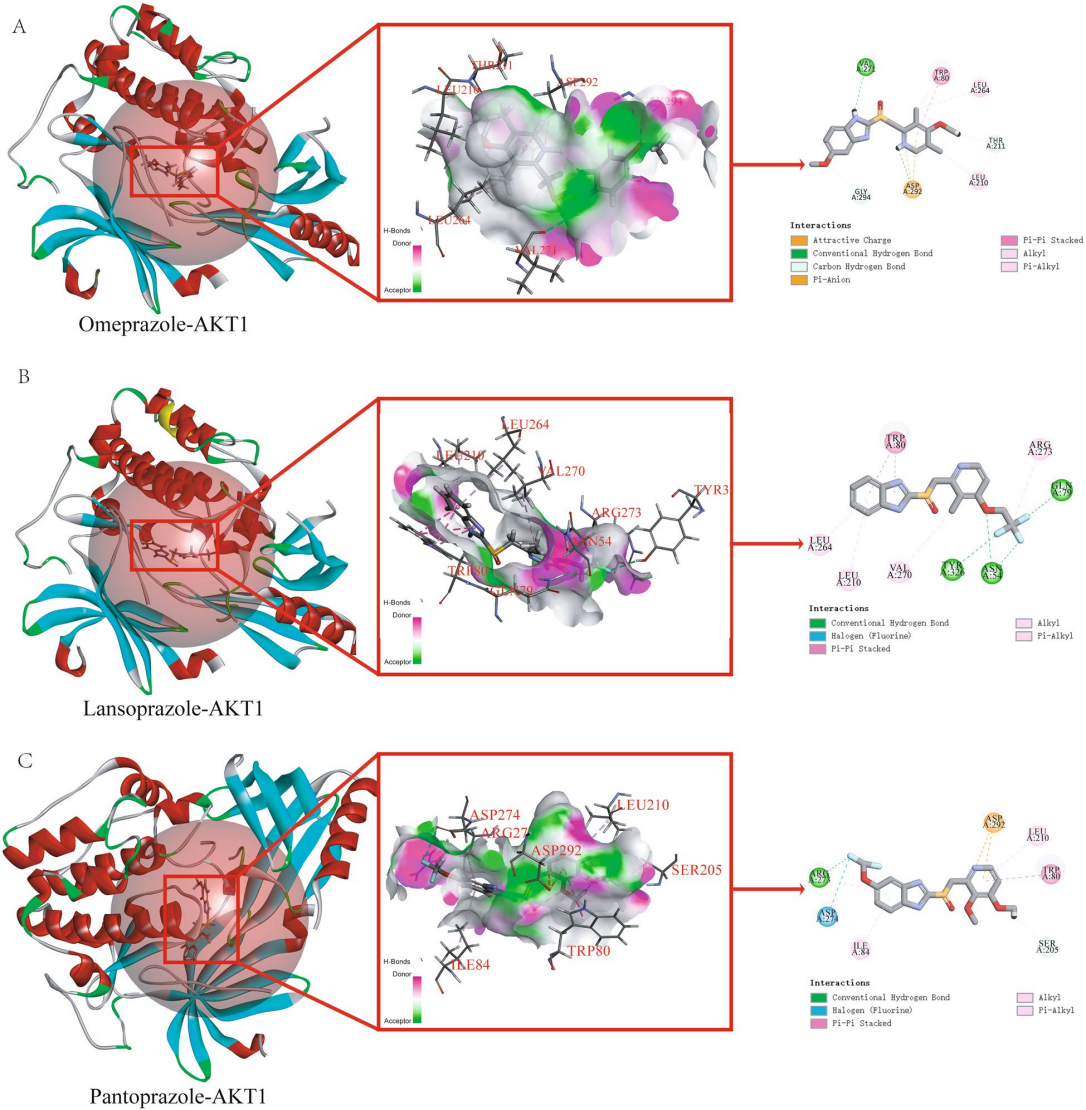
Molecular docking results of proton pump inhibitors and AKT1. (**A**) Molecular docking results of omeprazole and AKT1. (**B**) Molecular docking results of lansoprazole and AKT1. (**C**) Molecular docking results of pantoprazole and AKT1.

**Figure 7:**
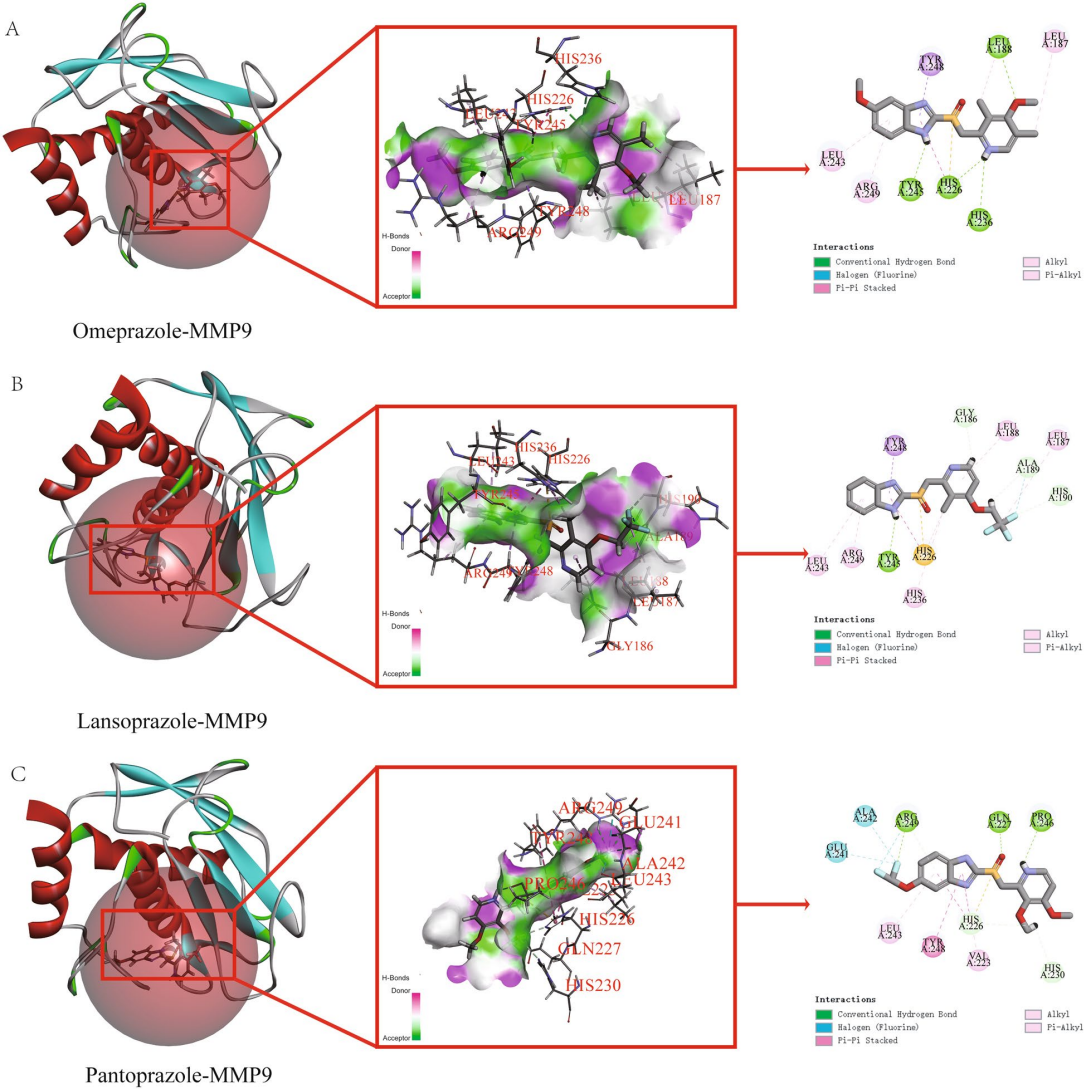
Molecular docking results of proton pump inhibitors and MMP9. (**A**) Molecular docking results of omeprazole and MMP9. (**B**) Molecular docking results of lansoprazole and MMP9. (**C**) Molecular docking results of pantoprazole and MMP9.

We performed molecular docking to investigate the binding affinity of *AKT1* and *MMP9* with proton pump inhibitors. The CDOCKER algorithm in Discovery Studio software was used for docking and the results were shown in Table 4. The binding free energies of omeprazole, lansoprazole, and pantoprazole after docking with *AKT1* were − 102.2277 kcal/mol, − 32.7706 kcal/mol, and − 93.1056 kcal/mol, respectively (Fig. 6A–C). After docking with *MMP9*, the binding free energies were − 115.1199 kcal/mol, − 53.7624 kcal/mol, and − 126.0212 kcal/mol for omeprazole, lansoprazole, and pantoprazole, respectively (Fig. 7A–C). According to the widely accepted criterion, small molecule ligands and proteins have good binding when the free energy of binding is less than − 7 kcal/mol. Our results showed that all three proton pump inhibitors had good binding affinities with both *AKT1* and *MMP9*, with binding free energies much lower than − 7 kcal/mol. These results suggested that *AKT1* and *MMP9* could be potential therapeutic targets for proton pump inhibitors in the treatment of diseases, especially for omeprazole and pantoprazole. Further studies are needed to explore the underlying mechanisms and clinical implications of these findings.

**Table 4:** The result of molecular docking with AKT1 and MMP9.

Receptor	Grid box	Omeprazole (kcal/mol)	Lansoprazole (kcal/mol)	Pantoprazole (kcal/mol)
AKT1 (PDB: 4ejn)	X = 35.389442	− 102.2277	− 32.7706	− 93.1056
	Y = 43.721140			
	Z = 18.443372			
	Ra = 15.743137			
MMP9 (PDB: 6esm)	X = 1.781333	− 115.1199	− 53.7624	− 126.0212
	Y = 50.977267			
	Z = 19.670200			
	Ra = 11.258874

## Discussion

Breast cancer and diabetes are two diseases that affect millions of people worldwide. Recent studies have shown a correlation between them, with diabetes being a high-risk factor for breast cancer^30^. In cancers with diabetes, high levels of insulin increase glucose uptake by cancer cells, which further promotes aerobic glycolysis for energy^9,31^. Therefore, drugs that target insulin signaling pathways or glucose metabolism may have potential in treating both diabetes and breast cancer. Additionality, chemotherapy drugs used to treat breast cancer can have an impact on a patient's blood sugar levels, especially for those who have pre-existing diabetes or impaired glucose metabolism. The drugs can interfere with the body’s metabolic processes, causing fluctuations in blood sugar levels and potentially worsening diabetes symptoms^32,33^. Chemotherapy drugs can also cause a decrease in insulin production or insulin resistance, which can lead to higher blood sugar levels^34^. This can be particularly problematic for patients with diabetes who are already at risk of developing complications such as nerve damage, cardiovascular disease, and kidney damage. Hence, there is a need for effective treatment options that can address both diseases. Nevertheless, due to the complexity of their underlying mechanisms, there is no single drug that can effectively treat both conditions. Fortunately, proton pump inhibitors (PPIs) have shown potential in treating cancer and diabetes due to their ability to inhibit vacuolar ATPases (V-ATPases) that play a role in lipid metabolism and intracellular pH regulation in cancer cells^35^. Specifically, PPIs can reduce the availability of lipids to induce cancer cell death^36^ and inhibit ATPase activity in the pancreas to regulate insulin production and increase insulin sensitivity^37,38^. However, the exact mechanism of PPIs in breast cancer with diabetes is still unclear, and further research is needed to fully understand their effects.

To investigate the potential targets and mechanisms of proton pump inhibitors that exhibit dual effects of anti-cancer and hypoglycemic, we chose three types of proton pump inhibitors including omeprazole, lansoprazole, and pantoprazole as our research subjects and utilized network pharmacology research methods. We obtained a total of 1838 targets for breast cancer and 417 targets for diabetes from the GeneCards database using a relevance score of ≥ 10. The predicted targets of the PPIs were intersected with the disease targets, resulting in the identification of 33 common targets of PPIs and the two diseases. Gene ontology (GO) and Kyoto Encyclopedia of Genes and Genomes (KEGG) enrichment analyses were performed to explore the biological processes and pathways involved in the identified genes. We found that the therapeutic targets were mainly enriched in fatty acid transport, cell proliferation, regulation of apoptotic signaling, metabolic processes of small molecules, responses to reactive oxygen species, and the activity of nuclear receptors, ligand − activated transcription, and nuclear hormone receptors. The KEGG pathway analysis revealed several significantly enriched pathways, including prostate cancer, diabetic cardiomyopathy, lipid and atherosclerosis, and insulin resistance. From the enrichment results, we know that intersection genes are closely related to the fatty acid transport. As the first link of fatty acid metabolism, fatty acid transport plays a key role in subsequent physiological processes. Yang et al.^39^ showed that blocking fatty acid intake could significantly inhibit the occurrence and development of breast cancer. As an important pathway of oxidative energy supply in breast cancer cells, fatty acid metabolism may become a potential therapeutic target for cancer therapy. In addition, we found an interesting phenomenon in which insulin resistance was also a major component of functional enrichment. Fatty acids induce insulin resistance, which is a hallmark of diabetes^40^. The Lipid and atherosclerosis, diabetic cardiomyopathy and other signaling pathways related to diabetes were also significantly enriched in our results. These results suggest that fatty acids may be a potential target for the treatment of breast cancer complicated with diabetes. To further identify the targets of proton pump inhibitors (PPIs), a protein–protein interaction network had constructed using the 33 intersection genes mentioned above. After constructing the regulatory network in Cytoscape software, we used the CytoNCA tool to calculate various network centrality measures for each node, resulting in six core targets: *AKT1, MMP9, PPARG, MMP2, KDR,* and *ALB*. We analyzed the expression levels of these targets in breast cancer and normal tissues and found that only *AKT1* and *MMP9* were significantly upregulated in breast cancer tissues compared to normal tissues, suggesting that they may be the most promising targets for proton pump inhibitors. Finally, we performed molecular docking to investigate the binding affinity of *AKT1* and *MMP9* with PPIs. We found that all three PPIs had good binding affinities with both *AKT1* and *MMP9*, with binding free energies much lower than − 7 kcal/mol. These results suggest that *AKT1* and *MMP9* could be potential therapeutic targets for PPIs in the treatment of diseases, especially for omeprazole and pantoprazole. However, further studies are needed to explore the underlying mechanisms and clinical implications of these findings.

The article has some limitations that need to be addressed. Firstly, the study only investigated three types of proton pump inhibitors, which may not be representative of all PPIs available in the market. Secondly, the study used a network pharmacology approach, which relies on the accuracy and completeness of the databases used to obtain the target genes. There is a possibility of missing some important genes that may be involved in the dual effects of PPIs. Thirdly, the study only focused on breast cancer and diabetes, and the results may not be applicable to other types of cancer or metabolic disorders. Finally, although the study identified six core targets of PPIs that may be potential therapeutic targets, further experimental validation is required to confirm their role in the dual effects of PPIs. Despite these limitations, the study provides valuable insights into the potential targets and mechanisms of PPIs that exhibit dual effects of anti-cancer and hypoglycemic and highlights the importance of fatty acid transport and insulin resistance in these effects.

In summary, this study sheds light on the potential therapeutic applications of proton pump inhibitors (PPIs) for breast cancer and diabetes. The research suggests that PPIs may have promising effects on *AKT1* and *MMP9*, and that omeprazole and pantoprazole may be effective in treating these diseases. The study also identified several biological processes and pathways that may be involved in the action of PPIs, as well as the regulatory network and molecular docking analyses that support the clinical utility of *AKT1* and *MMP9* as therapeutic targets. However, further research is required to confirm these findings and explore the underlying mechanisms. Overall, these results have important implications for the development of novel therapeutic strategies for breast cancer and diabetes.

## Data Availability

Data in this study can be obtained from the corresponding author upon reasonable request.

## Abbreviations

PPIs: Proton pump inhibitors
V-ATPase: Vesicular ATPase
GO: Gene ontology
KEGG: Kyoto Encyclopedia of Genes and Genomes
BP: Biological processes
CC: Cellular components
MF: Molecular functions
RMSD: Root mean square deviation
AKT1: V-akt murine thymoma viral oncogene homolog 1
MMP9: Matrix metalloproteinase 9
PPARG: Peroxisome proliferator-activated receptor gamma
MMP2: Matrix metalloproteinase 2
KDR: Kinase insert domain receptor
ALB: Albumin
